# DFT / TDDFT insights into excited state intra-molecular hydrogen atom transfer mechanism in Liqcoumarin: an EFP1 study

**DOI:** 10.3906/kim-2108-64

**Published:** 2021-10-25

**Authors:** Kandigowda JAGADEESHA, Yelechakanahalli Lingaraju RAMU, Mariyappa RAMEGOWDA

**Affiliations:** Department of Physics, Government College, Mandya, India

**Keywords:** Liqcoumarin, hydrated liqcoumarin, density functional theory, time dependent density functional theory, effective fragment potential

## Abstract

The theoretical calculations were carried out on Liqcoumarin (LC) and its water complex LC+(H_2_0)_4_-[LCH] at the ground (S_0_) / first excited states (S_1_) by employing density functional theory (DFT) / state specific time-dependent density functional theory (SS-TDDFT). In LC and LCH, there is an intra-molecular hydrogen bond between hydroxyl group and acetyl group along with four inter-molecular hydrogen bonds in the hydrated molecule. The computational studies of molecular structural parameters, electrostatic potential, NBO analysis, molecular orbital’s, and UV-Vis spectra of both molecules under polar solvents were explored by B3LYP / 6–31G (d,p) / PCM / EFP1 method. The intra-molecular hydrogen atom transpires between hydroxyl to acetyl group in both pure andhydrated molecules confirm the ICT process, and it affirms through potential energy surface (PES) scans.

## 1. Introduction

The phenomenon of hydrogen bonding has been illustrious for its eminence in various fields like photophysics,photochemistry, and biochemistry. The systematic study of hydrogen bonds is much essential for comprehending thephoto-physical properties of some organic molecules, which is having electron donor and acceptor demeanor at bothground and excited states [1–4]. The EFP method furnishes a polarizable QM-based force field to characterize the inter-molecular interactions. To study the microsolvation effect on biological or organic molecule, EFP1 / DFT method wasestablished explicitly for solvation, and it interfaced with the polarizable continuum model (PCM) [5,6], and also EFP1/ TDDFT method has been fostered for characterizing the electronically excited states of solvated molecules [5, 7–9]. Inorder to enlighten the electronic, molecular, structural, and photo-physical properties of many organic and biologicalmolecules, TDDFT computations are done at excited states. At the excited state, there is a refashioning in the molecularstructure, which takes place within the molecule along with hydrogen transfer. The revamp of hydrogen bond anatomydue to microsolvation effect is to evince the ESIPT / ESIHT process in some organic and biological molecules. Theintra-molecular charge transfer character correlated with ESIPT / ESIHT is associated to the photophysical features ofbiologically active molecules [10].

The naturally occurring herb Glycyrrhiza glabra (Licorice) is one of the most widely used herbs, which exhibits a broad range of biological activities both in traditional medicine and flavouring, belonging to the Leguminoseae (Fabaceae) family [11,12]. The roots of Glycyrrhiza glabra gave more than 400 compounds, and the main phenols identified from the root are isoliquiritin, liquiritin, and coumarins, including umbelliferone, Liqcoumarin (LC), glabro coumarone A and B [13–15]. Pharmacological studies have confirmed that biologically active compounds in Glycyrrhiza plant exhibit a broad range of biological activities like anti-diabetic [16], anti-microbial [17], anti-carcinogenic [18], anti-oxidant [19], cytotoxic, anti-tumor [20], anti-inflammatory [21,22], anti-viral [23]. A series of compounds synthesized from 6-coumarin derivatives showed potent inhibitory activity against human immunodeficiency virus (HIV-1) infection with IC_50_ [24]. The α-pyrano chalcones [Gb1–Gb2], which were synthesized from 6-acetyl-5-hydroxy-4-methyl-2H-chromen-2-one exhibits anti-malarial activity against P. Falciparum such that halogenated derivatives (Gb1) exhibited greater activity than the methoxylated derivative (Gb2) [25]. LC is one of the hydroxycoumarin having 26 atoms in which methyl (-CH3), hydroxyl (-OH), and acetyl groups are attached to the C4, C5, and C6 positions of basic coumarin moiety. LC has an intra-molecular hydrogen bond between hydroxyl group and acetyl group and can also form four inter-molecular hydrogen bonds with four solvent molecules. In the present theoretical study, the DFT / TDDFT / EFP1 methods were used to investigate LC and LCH molecules in the ground and excited states and also the electronic structure; ICT states of the molecules along with the ESIHT process have been explored.

## 2. Computational methods

Avogadro[26] is an advanced molecule editor and visualizer employed to design the pure molecule and its water complex, LCH, optimized at equilibrium geometry using MMFF94s force field [27]. All theoretical computations were executed by GAMESS-US software suite [28,29]. The gas phase geometry optimization of LC and LCH molecules at both ground state (S_0_) and first excited state (S_1_) was carried out without symmetry constraints using 6–31G(d,p) basis set [30]. This procedure was considered satisfactory if the energy difference between optimized cycles was (3–6) × 10^−5^ Hartree / Bohr and a gradient of <1×10^−5^ Hartree/Bohr was achieved. Computational methods like DFT [31–36] / SS-TDDFT [37,38] are the attractive and useful tools to generate the gas phase optimized molecular geometries of LC and LCH both at S_0_ and S_1_ states by using hybrid functional B3LYP [39,40] with 6–31G(d,p) basis set. The natural charges on various atoms and groups of both molecules at ground and excited states were computed using NBO.6 program [41]. The Frontier molecular orbitals and MEP maps are plotted by wxMacMolPlt[42], which is an open-source application for visualization.

## 3. Results and discussion

### 3.1. Electronic structural studies at S_0_ and S_1_ states

The gas phase optimized geometrical parameters of LC and LCH molecules at S_0_ and S_1_ states namely bond lengths, bond angles are computed by DFT / TDDFT / B3LYP / 6–31G(d,p) levels. At ground state of LC and LCH molecules, the intra-molecular HB O15-H23·····O16=C11 exist between hydroxy group and acetyl group and it modified as O16-H23·····O15=C1 at the first excited state. Four inter-molecular hydrogen bonds exist between the pure molecule and solvated water molecules; two HB’s will exist between oxygen of hydroxyl group and oxygen of carbonyl group and another two HB’s will exist across the keto group of pyrone ring. One hydrogen bond exists between micro-solvated water molecules across the hydroxyl group and oxygen of the carbonyl group.

When pure molecule LC undergo photo-excitation, the bond lengths C1-C2 / C3-C4 / C5-C6 / C7=C8 / C1-C6 increased by 0.026 / 0.044 / 0.053 / 0.032 / 0.034 Å and C2-C3 / C4=C5 / C6-C7 / C11-C12 / C2-C11 decreased by 0.021 / 0.038 / 0.044 / 0.024 /0.019Å. In the pyrone ring, bond lengths C9=O13 / C5-O14 increases by 0.01 / 0.011Å, whereas bond length C9-O14 decreased by 0.022 Å. Due to hydrogen atom transfer (H23) from hydroxyl group to the carbonyl group, C1-O15 becomes C1=O15, and its bond length decreased by 0.058Å; C11=O16 becomes C11-O16, and its bond length increased by 0.086Å. The bond lengths of C-H combinations in the LC molecule decreased / increased in the range of (1–5) ×10^−3^ Å. The bond angles between different atoms vary from 0.1^0^ to 4.6 ^0^

Due to the effect of micro-solvation, the optimized geometrical parameters of LCH molecule changes. The bond lengths C3-C4 / C7=C8 / C7-C10 / C2-C11 / C1-O15 increased by 0.004 / 0.002A / 0.001 / 0.006 / 0.01Å and C1-C2 / C2-C3 / C4-C5 / C5-C6 / C6-C7 / C8-C9 / C11-C12 / C11-C16 / C1=C6 decreased by 0.007 / 0.005 / 0.005 / 0.001 / 0.002 / 0.005 / 0.008 / 0.002 / 0.001 ×10^−2^Å. In the pyrone ring, bond lengths C9=O13 / C5-O14 / C1=O15 / O15-H23 increased by 0.011 / 0.008 / 0.01 / 0.012Å, whereas bond length C9-O14 / C11-O16 decreases by 0.014 / 0.002Å. The bond lengths of C-H combinations in the LCH molecule decreased / increased in the range of (0–6) × 10^−3^Å. The bond angles between the respective atoms varies from 0^0^ to 1.3^0^.

**Figure 1:**
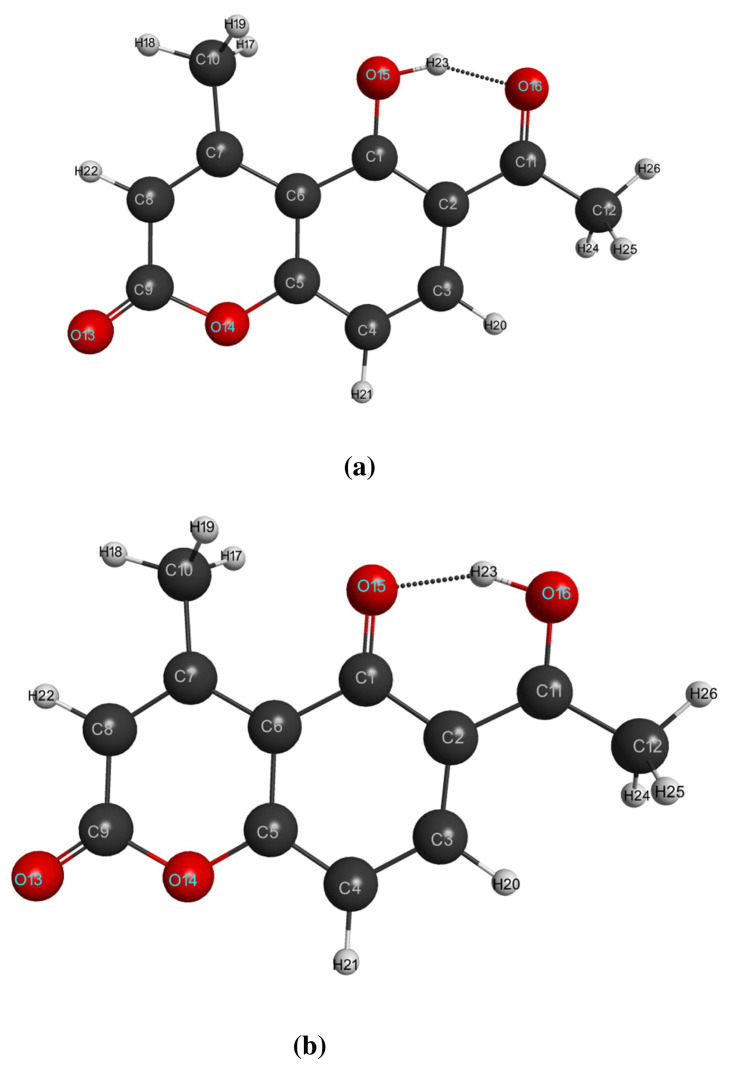
Optimized equilibrium molecular structures of LC molecule at (a) S_0_ and (b)S_1_ state.

**Figure 2:**
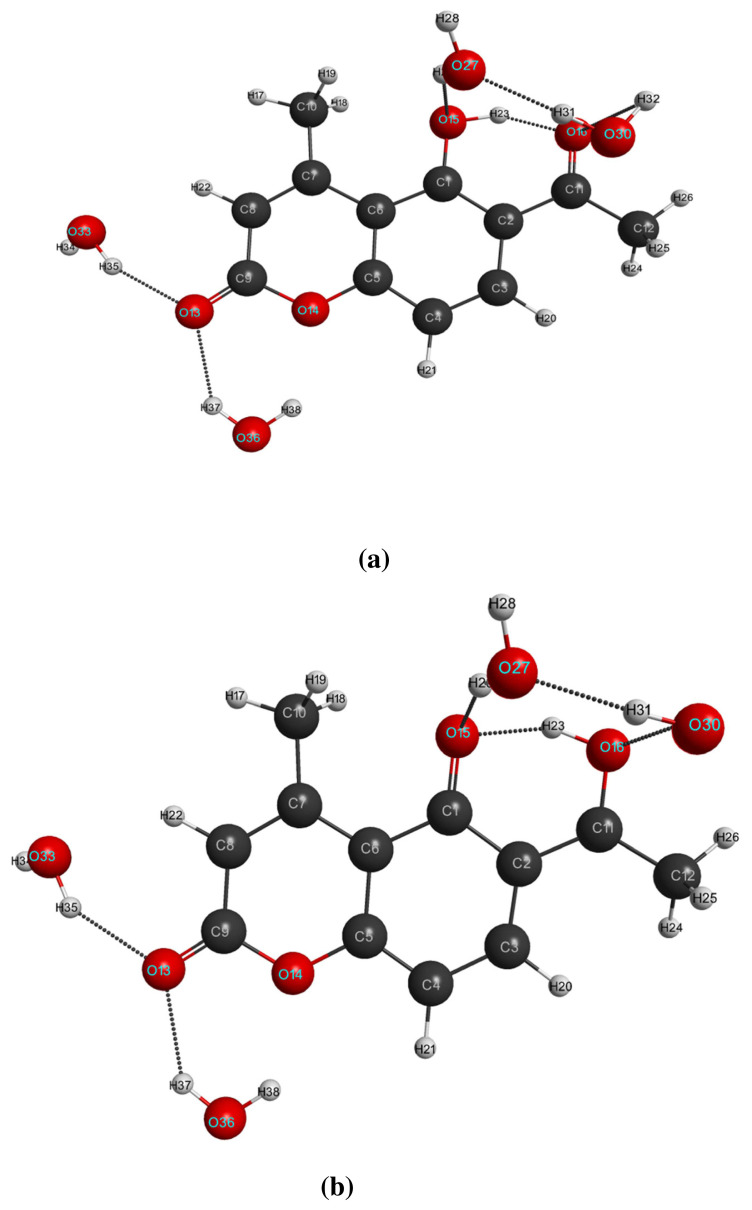
Optimized equilibrium molecular structures of LCH molecule at (a) S_0_ and (b)S_1_ state.

When microsolvated molecule LCH undergo excitation, the bond lengths C1-C2 / C3-C4 / C5-C6 / C7=C8 / C1=C6 increased by 0.029 / 0.043 / 0.049 / 0.0321 / 0.034 Å and C2-C3 / C4=C5 / C6-C7 / C11-C12 / C2-C11 decreased by 0.018 / 0.036 / 0.04 / 0.019 / 0.02Å. In the pyrone ring, bond lengths C9=O13 / C5-O14 increases by 0.004 / 0.01Å, whereas bond length C9-O14 decreased by 0.015Å. Due to hydrogen atom transfer (H23) from hydroxyl group to carbonyl group, C1-O15 becomes C1=O15, and its bond length decreased by 0.061Å; C11=O16 becomes C11-O16, and its bond length increased by 0.092Å. The bond lengths of C-H combinations in the LC molecule decreased / increased in the range of (1–6) × 10^−3^Å. The bond angles between different atoms vary from 0.1 ^0^ to 3.8^0^. The optimized equilibrium structures of LC and LCH molecules at S_0_ and S_1_ states with an atom numbering scheme are shown in Figures 1,2. The structural parameters like bond lengths, bond angles at S_0_ and S_1_ states are summarized in Tables 1,2.

### 3.2. Simulation of UV-Vis spectra

To our knowledge, theoretical studies on LCH have been performed so far to study its interaction in water and methanol solvents. It is observed that, the bond lengths, bond angles, dihedral angles of LC and LCH molecules change appreciably, and also excitation energy in the first excited state changes due to the non-covalent bonding interaction between LC and LCH with polar solvents, i.e. the first excitation energy of LC and LCH molecules corresponding to methanol and water solvents as 3.788 eV / 3.872eV, 3.789eV / 3.871eV, while the excitation energy of LC and LCH molecules in the gas phase is 3.756eV / 3.901eV.

**Figure 3:**
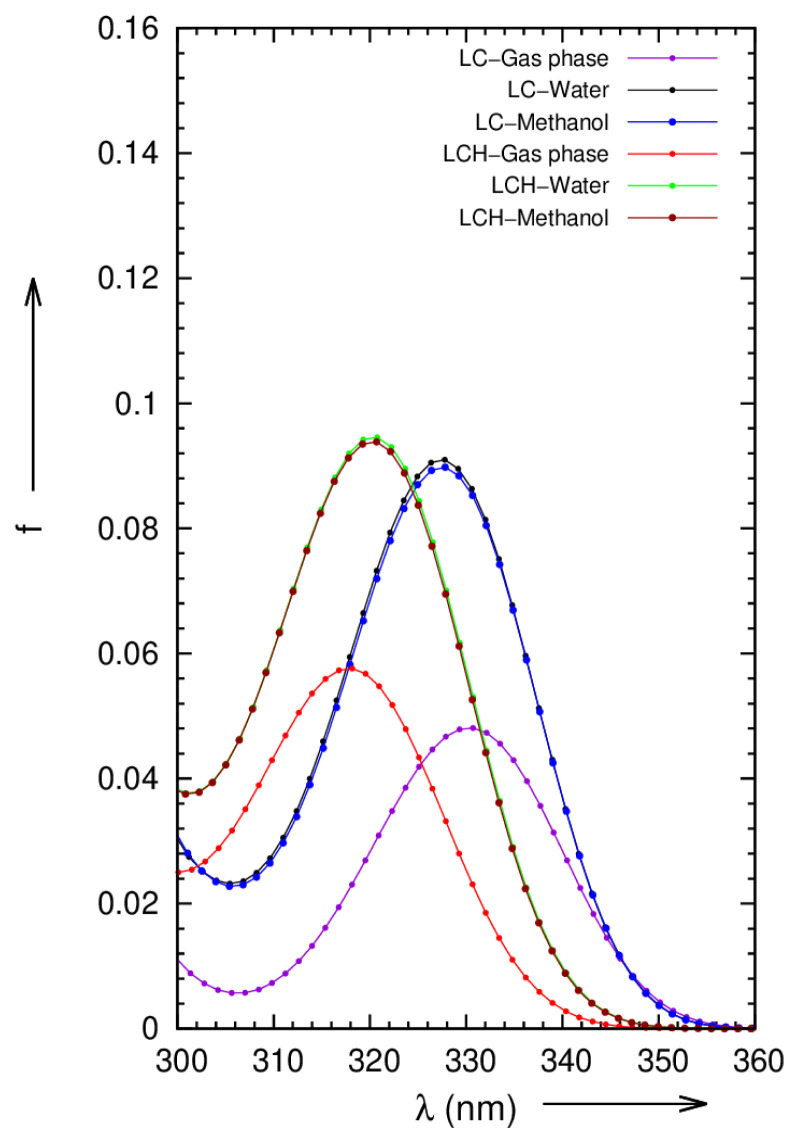
Simulated absorption spectra of LC and LCH molecules in gas phase, water and methanol solvents.

The electronic transitions that occur in the pure and micro solvated molecules LC and LCH are identified by UV-Vis spectral analysis. By using TDDFT / PCM / B3LYP / 6–31G (d, p) method, the theoretical electronic absorption spectral wavelengths of LC and LCH molecules in the gas phase / water / methanol exhibit an intense band near 330 / 327.2 / 327.3nm, and 317.8 / 320.29 / 320.21nm, respectively. The calculated electronic absorption spectra of LC and LCH in the gas phase and in various polar protic solvents using the polarizable continuum model (PCM) are presented in Figure 3. The locality of absorption bands is regulated by water and methanol solvents. The vertical absorption energies, oscillator strengths, and probable wave functions are depicted in Table 3 evinces solvent effects on the electronic π→π* transitions states of LC and LCH molecules, i.e. in polar solvents; the energies of the π→π* electronic transitions are increased and thereby decrease in absorption wavelength; the UV-Vis spectra signalize a systematical blue shift in LCH, and the calculated absorption wavelength difference corresponds to 12.2nm, 6.91nm, and 7.09nm respectively, while moving from gas to methanol solvents. The theoretical absorption spectral wavelength of pure LC in methanol solvent was about 327.3 nm, which is approximately consistent with experimental value, which is about 310nm [43].

### 3.3. Molecular orbitals

**Figure 4:**
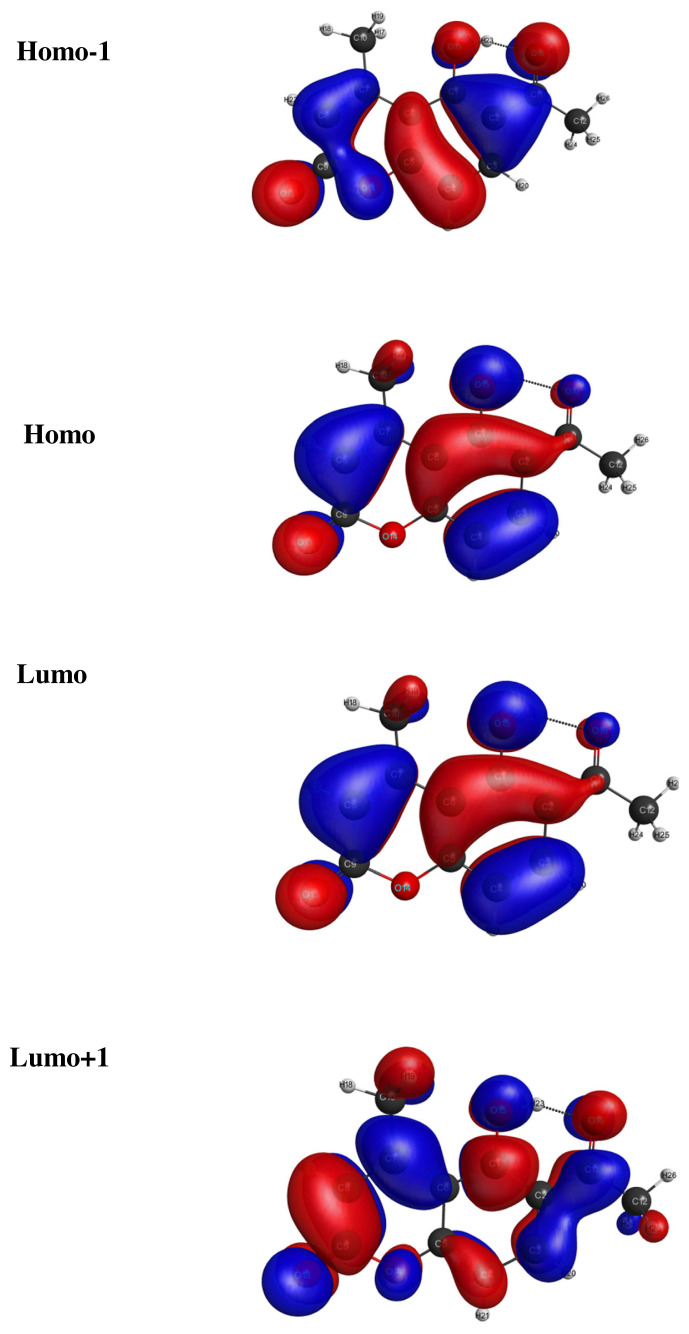
The molecular orbitals of LC molecule.

**Figure 5:**
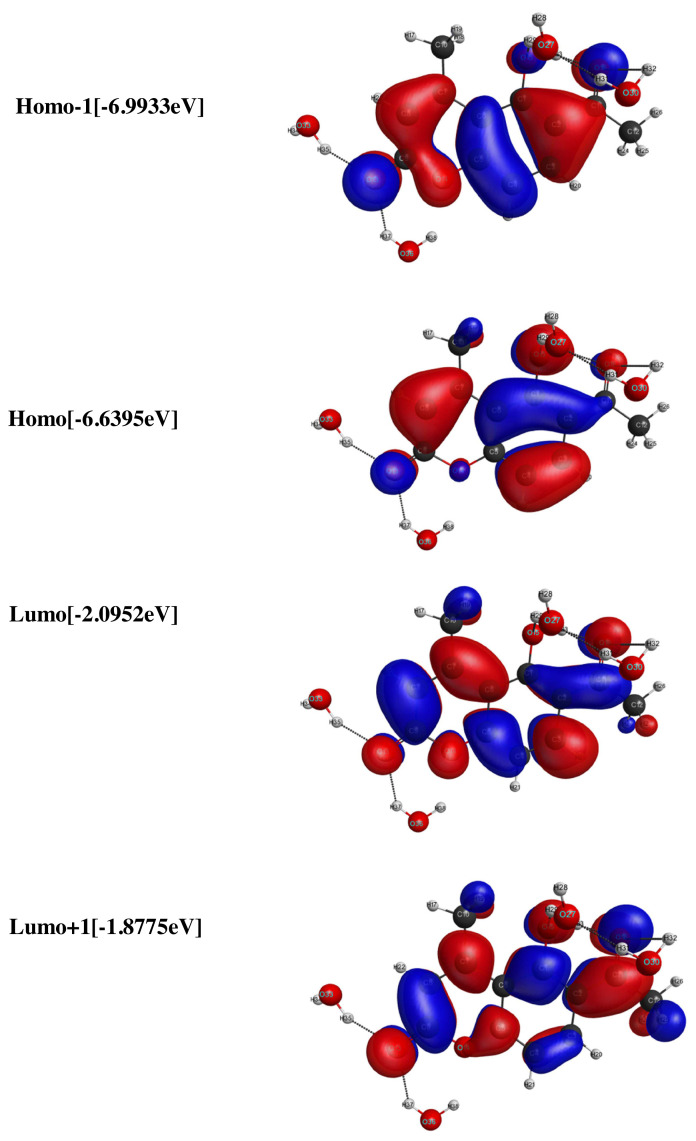
The molecular orbitals of LCH molecule.

The frontier molecular orbitals are discussed for both LC and LCH molecular systems. HOMO, HOMO-1, LUMO and LUMO+1 energies were computed by TD-DFT / B3LYP / 6–31G(d,p) method and are picturized in Figures 4,5. The LUMO+1, LUMO, HOMO and HOMO-1 energies corresponds to LC / LCH molecules are −1.5782eV / −1.8775eV, −1.9592eV / −2.0952eV, −6.3402eV / −6.6395eV and −6.6667eV / 6.9933eV. The energy gap between (HOMO-LUMO), (HOMO-1-LUMO+1) explains the eventual charge transfer reactions occur within the LC / LCH molecules, which are given by 4.381eV, 5.088eV / 4.5443eV, 5.11158eV. It is observed that, the energy difference between (HOMO-LUMO) and (HOMO-1-LUMO+1) increases in LCH due to the effect of microsolvation.

### 3.4. Natural charge analysis

The NBO 6.0 calculations were carried out at both ground and first excited states of LC and LCH using computational package like GAMESS integrated with NBO 6.0 program at DFT / TDDFT / B3LYP / 6–31G(d, p) level. It provides a convenient basis for investigating charge transfer mechanism in LC and LCH molecules at first excited state. The charge analysis rendered on the electronic structure of LC and LCH molecules precisely describes the allocation of electrons in different sub shells of their AOs. The natural charges on various atoms of LC and LCH molecules at ground and excited states are tabulated in Table 4. The natural charges on O15 / O16 / H23 atoms becomes −0.694e / −0.601e / 0.527e and −0.736e / −0.614e / 0.527e, respectively in the ground state of LC and LCH molecules. The total natural charge of acetyl / hydroxyl group (O15-H23) / methyl groups / benzene ring / pyrone rings becomes 0.015e / −0.167e / 0.073e / 0.435e / −0.115e and 0.039e / −0.209e / 0.07e / 0.465e / −0.157e in the ground state of LC and LCH molecules, respectively. The natural charge on O15 / O16 / acetyl group / hydroxyl group / benzene ring / pyrone ring increased by −0.042e / −0.012e / 0.048e / 0.054e / 0.030e / −0.042e, where as natural charge on H23 / methyl group decreased by 0.0005e / 0.0027e due to effect of micro-solvation.

When LC and LCH molecules undergo photo-excitation, the natural charges on O15 /O16 / H23 atoms become −0.667e / −0.666e / 0.523e and −0.716e / −0.684e / 0.528e respectively. The total natural charge of methyl group / benzene ring / pyrone rings becomes 0.094e / 0.484e / 0.059e and 0.096e / 0.532e / 0.032e in LC and LCH, respectively. The natural charge on O16 / methyl group / benzene ring / pyrone ring increased by −0.065e / −0.021e / 0.049e / 0.173e, −0.07e / 0.026e / 0.067e / 0.189e, whereas natural charge on O15 / H23 decreased by −0.027e / 0.004e, −0.020e / 0.005e in the first excited state of LC and LCH molecules. In LC molecule, the charge on O15 / H23 decreased by 0.027e / 0.004e, and the charge on O16 increased by 0.065e. Whereas, in LCH molecule, the charge on O15 / H23 decreased by −0.02e / 0.005e, and the charge on O16 increased by 0.07e. So, an intra-molecular hydrogen atom (H23) transfer takes place from hydroxyl group (O15-H23) to carbonyl group (C11-O16)., i.e ICT takes place both in LC and LCH molecules in the excited state.

### 3.5. Molecular electrostatic potential (MEP)

**Figure 6:**
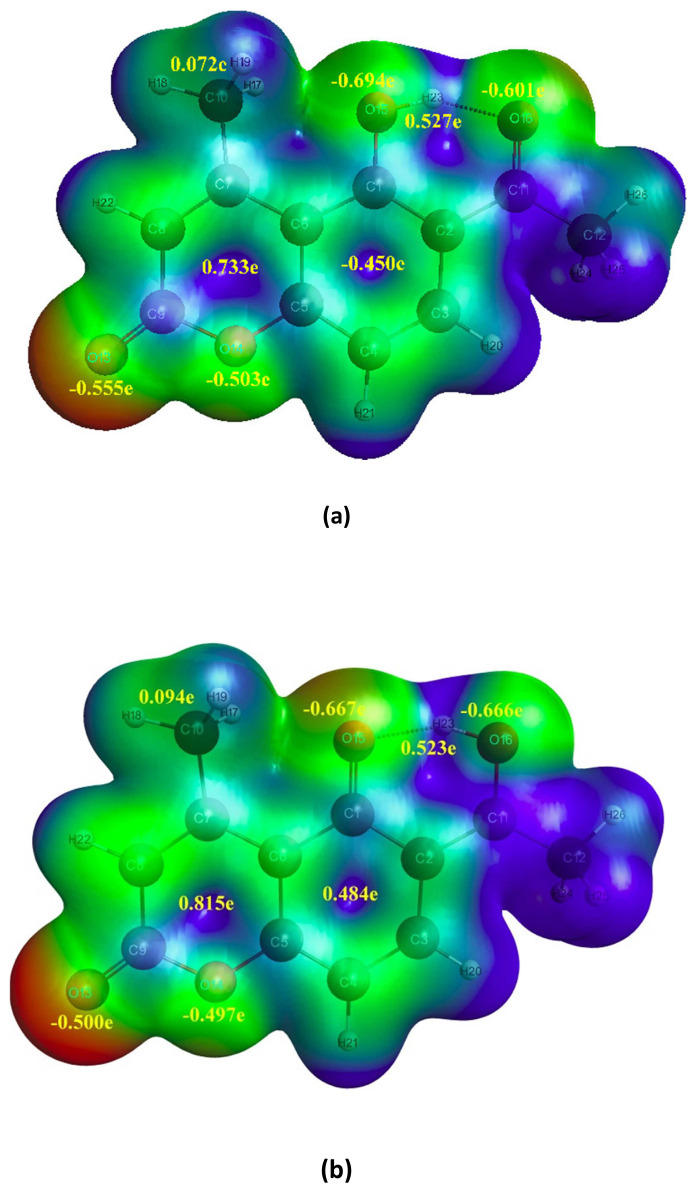
MEP with charges on different atoms and groups of LC molecule at (a)S_0_ and (b)S_1_ states.

**Figure 7:**
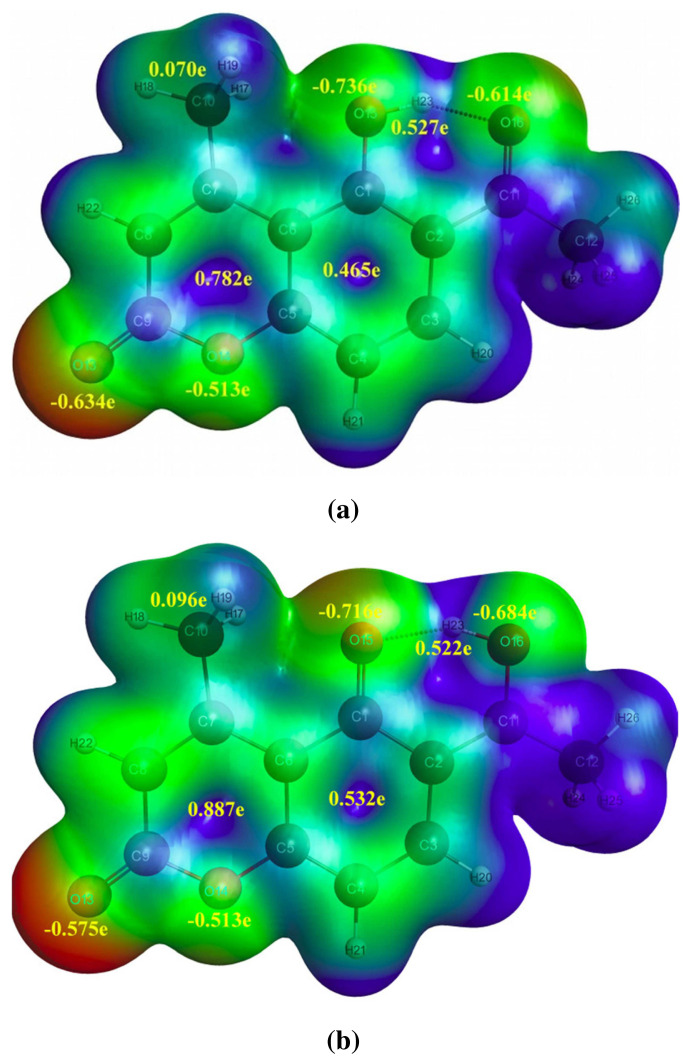
MEP with charges on different atoms and groups of LCH molecule at (a)S_0_ and (b)S_1_ states.

The 3D plot of total electron density called MEP is enlightened to predict reactive sites for the LC and LCH molecules by employing the optimized geometry parameters at 6–31G(d,p) / B3LYP / DFT level. MEP can be a substantial presentation for the elucidation of non-covalent interactions, aggression of electrophilic and nucleophilic at appropriate zone of the investigated system. The magnitude of the electrostatic potential at the LC and LCH are expressed by benchmark colors, i.e. red, blue and green colors displays the most negative, positive, and zero electrostatic potential. From MEP of LC and LCH molecules at the ground and first excited states, the dark red colored zone over the oxygen atoms delineates electrophilic (electron rich) reactivity, and the dark blue coloured zone over the hydrogen atoms delineates the nucleophilic (electron deficient) reactivity, i.e. the sites insinuating the negative electrostatic potential are focalized at the conjugated aromatic rings, whereas the sites insinuating the positive potential are focalized at the aromatic hydrogen atoms. The MEP plots with charges on various atoms and groups of LC and LCH molecules at ground and first excited states are portrayed in Figures 6,7.

### 3.6. Hydrogen bond (HB) dynamics

To depict the comprehensive perspectives of both intra-molecular and inter-molecular hydrogen bonds at ground state and first excited states, theoretical study have been effectuated on pure molecule and its water complex at DFT / TDDFT level by enforcing 6–31G(d,p) / B3LYP method. At ground state of LC / LCH molecules, an intra-molecular HB O15-H23·····O16 = C11of length 1.569 / 1.520Å and dihedral angle −0.3° / 4.2° will be formed between hydroxyl and acetyl group. But, at the excited state, this intra-molecular HB is modified as O16-H23·····O15 = C1 of length 1.571 / 1.599 Å and dihedral angle 0.7° / −10.6°. In LCH molecule, four inter-molecular hydrogen bonds were formed; two were formed across 2-pyrone of the coumarin moiety, and another two inter-molecular hydrogen bonds formed between the hydroxyl group and acetyl group with one Hydrogen bond were formed between water molecules.

In the S_0_ state of LCH, inter-molecular hydrogen bonds like C9=O13·····H35-O33 / C9=O13·····H37-O36 formed across C9=O13 of coumarin moiety of their bond lengths 1.872 / 1.979Å and dihedral angles 19.2^0^/ −0.2^0^. The other two inter-molecular hydrogen bonds like C1-O15·····H29-O27 / C11=O16·····H32-O30 formed across O15 and O16 of hydroxyl group and acetyl group of their bond lengths 1.935 / 2.675Å and dihedral angles 48.7° / −36°. Whereas, in the S_1_ state of LCH, inter-molecular hydrogen bonds like C9=O13·····H35-O33 / C9=O13·····H37-O36 formed across C9=O13 of coumarin moiety of their bond lengths 1.924 / 2.002Å and dihedral angles 9.2°/ −1.5°. The other two inter-molecular hydrogen bonds like C1-O15·····H29-O27 / C11=O16·····H32-O30 formed across O15 and O16 of hydroxyl group and acetyl group of their bond lengths 1.865 / 2.070Å and dihedral angles 53.7° / −52.8°. One HB like O30-H31·····O27-H29 of length 1.814 / 1.843Å and dihedral angle 11.3° / 32.5° exist between two water molecules, which are bonded with hydroxyl group and acetyl group of LCH corresponds to S_0_ / S_1_ states.

In the first excited state of LC molecule, the charge on O15 / H23 decreased by 0.027e / 0.004e and the charge on O16 increased by 0.065e. While, in LCH molecule, the charge on O15 / H23 decreased by 0.020e / 0.005e and the charge on O16 increased by 0.07e. As the repercussion, the intra-molecular HB O15-H23·····O16=C11is recasts as O16-H23·····O15=C1 due to transfuse of hydrogen atom H25 from hydroxyl group (O15-H23) to carbonyl group (C11-O16). Similarly, in the first excited state of LCH molecule, the inter-molecular HB’s C9=O13·····H35-O33 / C9=O13·····H37-O36 elongated by 0.052 Å / 0.023Å due to decrease in charge on O13 and inter-molecular HB’s C1-O15·····H29-O27 / C11=O16·····H32-O30 contracted by 0.070Å / 0.605Å due to decrease / increase in charge on O15 / O16 by 0.020e / 0.070e. The hydrogen bond O30-H31·····O27-H29 exists between water molecules gets elongated by 0.039 Å. The intra-molecular and inter-molecular HB lengths of pure and hydrated molecules at S_0_ and S_1_ states are tabulated in Tables 5,6.

### 3.7. PES scan and ESIHT mechanism

**Figure 8:**
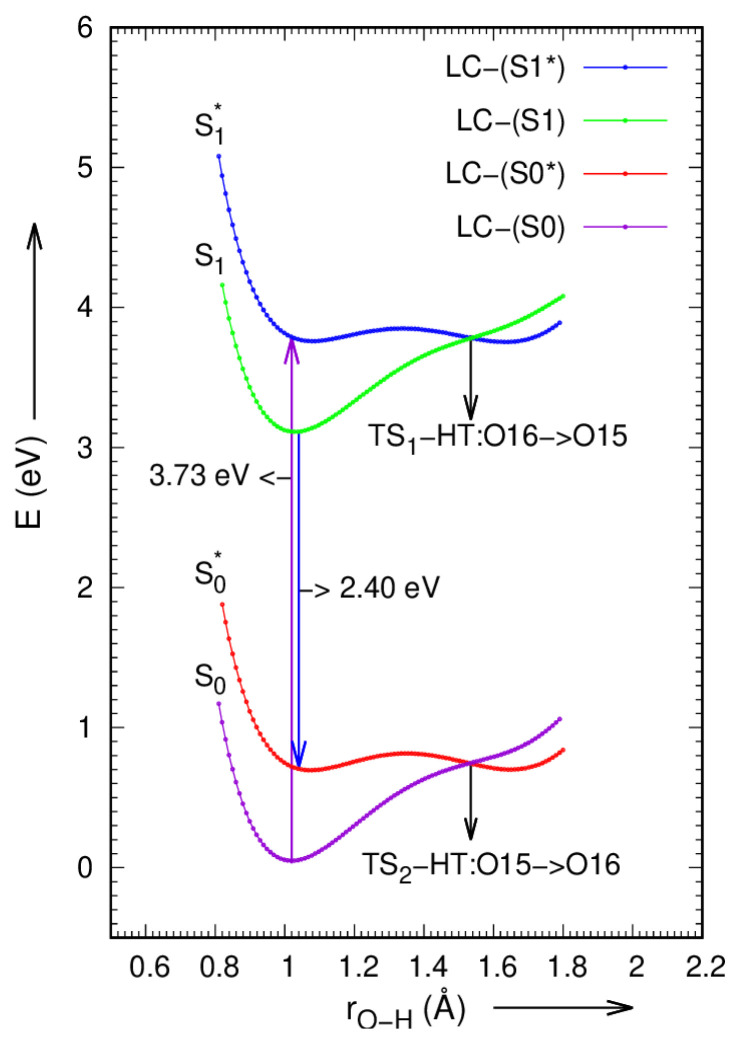
Potential energy plot along intra-molecular hydrogen transfer path in LC molecule.

**Figure 9:**
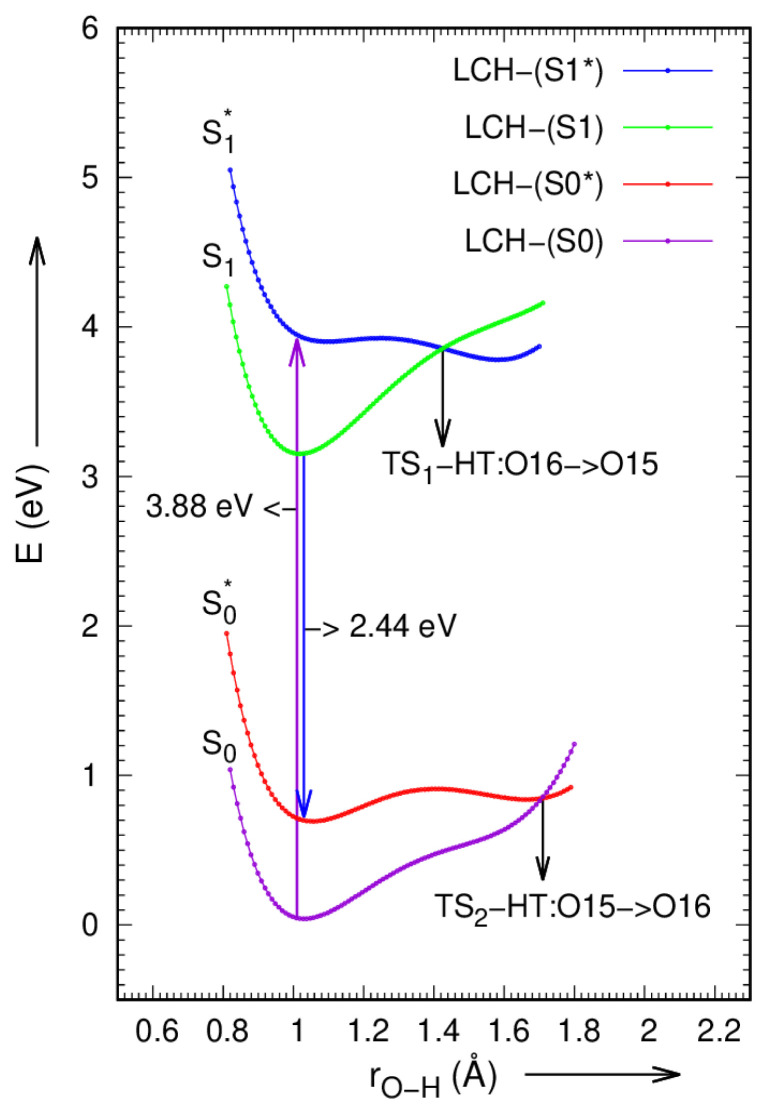
Potential energy plot along intra-molecular hydrogen transfer path in LCH molecule.

Envisaging and inspecting of PES curves is an efficient manner to investigate the ESIHT mechanism in LC and LCH molecules. Based on constrained relaxed and un-relaxed optimizations in both S_0_ and S_1_ states of LC and LCH molecules, potential energy curves have been scanned via intra-molecular HB O15-H23·····O16 = C1 by varying the O15-H23 and O16-H23 bond lengths from 0.8 to 1.8 Å in steps of 0.02 Å are presented in Figure 5. The ground and first excited state potential energy scans of LC and LCH molecules can be done in gas phase with DFT/TDDFT methods by using hybrid functional B3LYP / 6–31G(d,p) basis set. The LC and LCH molecules can persist in unrelaxed first excited state (S^*^_1_) by irradiating with the radiation of energy 3.73 eV and 3.88 eV, the O16-H23 bond extends to 1.535 Å and 1.425 Å respectively, the H23 atom isolated from O16 and embedded to O15. This is the junction at which ESIHT occurs. The LC and LCH molecules set in the relaxed first excited state S_1_ with O16-H23 bond length of 1.016Å and 1.008Å, respectively. The fluorescence may occur in both LC and LCH molecules with the emission of 2.40eV and 2.44eV energy; the molecules de-excited to unrelaxed ground state, S^*^_0_. At this condition, the O15–H23 bond length in LC and LCH molecules again elongates to 1.535Å and 1.71Å respectively, where the eviction of hydrogen atom (H23) from O15 to O16 occurs, and the molecules regress to their ground state. The hydrogen transfer mechanism in both LC and LCH molecules is professed by drawing potential energy surface scan maps, which are portrayed in Figures 8,9.

## 4. Conclusion

The theoretical studies of LC and LCH molecules at S_0_ and S_1_ states using the DFT / TDDFT / EFP1 method proclaim the molecular structure, inter and intra-molecular HBs. The studies of frontier molecular orbitals and the NBO calculation both in the ground and excited states assist the leaning of hydrogen atom transpires from hydroxyl group to the acetyl group due to intramolecular charge allocation escalating the acidity / basicity of the donor-acceptor units. The detailed investigation of the UV-Vis spectra and energy specification along the hydrogen transfer track in an excited state asserts the ESIHT mechanism in LC and LCH molecules. To conclude, the relaxation dynamics of excited LC and LCH can be controlled by the intramolecular charge allocation and the hydrogen-bonding geometry in the S1 state. This computational study affords the existent research on the chemical and biological activity of the liqcoumarin molecule.

## Figures and Tables

**Table 1:** Selected bond lengths of LC and LCH molecules at S_0_ and S_1_ states.

r(Å)	LC-S0	LC-S1	LCH-S0	LCH-S1
C1-C2	1.426	1.452	1.419	1.448
C2-C3	1.413	1.392	1.408	1.39
C3-C4	1.376	1.42	1.38	1.423
C4-C5	1.405	1.367	1.4	1.364
C5-C6	1.411	1.464	1.41	1.459
C6-C7	1.461	1.417	1.459	1.419
C7-C8	1.361	1.393	1.363	1.394
C8-C9	1.447	1.441	1.442	1.441
C9-O13	1.207	1.216	1.218	1.222
C5-O14	1.353	1.364	1.361	1.371
C9-O14	1.406	1.384	1.392	1.377
C7-C10	1.506	1.502	1.507	1.502
C8-H22	1.084	1.083	1.083	1.082
C10-H17	1.094	1.093	1.092	1.092
C10-H18	1.092	1.093	1.093	1.092
C10-H19	1.093	1.093	1.094	1.094
C12-H24	1.095	1.099	1.095	1.097
C12-H25	1.096	1.101	1.094	1.1
C12-H26	1.09	1.092	1.09	1.092
C11-C12	1.514	1.49	1.506	1.487
C2-C11	1.466	1.447	1.472	1.452
C11-O16	1.246	1.332	1.244	1.336
C1-O15	1.332	1.274	1.342	1.281
C15-H23	1.007	1.016	1.019	1.008
C1-C6	1.425	1.459	1.424	1.458

**Table 2:** Selected bond angles - A(°) of LC and LCH molecules at S_0_ and S_1_ states.

A(^0^)	LC-S0	LC-S1	LCH-S0	LCH-S1
C1-C2-C3	119.0	118.5	119.0	118.2
C2-C3-C4	121.5	124.4	121.5	124.3
C3-C4-C5	119.0	118.4	118.7	118.4
C4-C5-C6	123.0	121.4	123.2	121.5
C5-C6-C7	117.0	118.0	117.7	118.0
C6-C7-C8	118.3	118.2	118.6	118.5
C7-C8-C9	124.0	123.6	123.2	123.0
C8-C9-O14	115.2	117.0	116.2	117.6
C9-O14-C5	122.8	121.8	122.7	121.7
O14-C5-C6	122.0	121.5	121.5	121.4
C1-O15-H23	106.0	101.4	104.7	100.9
C2-C1-O15	120.0	120.7	119.8	120.7
C2-C11-O16	121.0	120.3	120.4	120.0
C1-C2-C11	119.0	119.6	119.0	120.0
C12-C11-O16	118.4	115.6	119.1	115.4
C6-C1-O15	119.0	121.1	119.2	120.6

**Table 3:** Peaks represents the theoretical simulated UV-Vis absorption wavelengths (λ) of LC and LCH molecules corresponds to electronic transition S_0_→S_1_ with oscillator strengths (f) in gas phase, water and methanol solvents.

Molecule	Solvent / gas phase	λ_a_ (nm)	f	Wave function (excitation amplitude)
**LC**	Gas phase	330.0	0.0557	H→L(−0.916), H-1→L(−0.141), H-1→L+1(−0.118) H→L+1(0.332), H-1→L+2(−0.130)
	Water	327.2	0.1065	H→L(−0.941), H-1→L(−0.130), H-1→L+1(−0.104) H→L+1(0.268), H-1→L+2(−0.120)
	Methanol	327.3	0.1053	H→L(0.943), H-1→L(0.127), H-1→L+1(0.103) H→L+1(−0.263), H-1→L+2(0.118)
**LCH**	Gas phase	317.8	0.0685	H→L(0.903), H-1→L(0.158), H-1→L+1(−0.102) H→L+1(0.360), H-1→L+2(0.138)
	Water	320.29	0.1139	H→L(0.937), H-1→L(−0.139), H-1→L+1(−0.098) H→L+1(−0.279), H-1→L+2(−0.123)
	Methanol	320.21	0.1130	H→L(−0.937), H-1→L(0.138), H-1→L+1(0.096) H→L+1(0.280), H-1→L+2(0.123)

**Table 4:** Natural charges on different atoms of LC and LCH molecules at S_0_ and S_1_ states.

Atomic number	LC-S0	LC-S1	LCH-S0	LCH-S1
C1	0.4424	0.4534	0.4350	0.4554
C2	−0.2602	−0.2618	−0.2407	−0.2537
C3	−0.1677	−0.1094	−0.1549	−0.0926
C4	−0.3039	−0.3493	−0.3000	−0.3283
C5	0.4015	0.3888	0.3920	0.3721
C6	−0.1903	−0.1472	−0.1843	−0.1387
C7	0.0616	0.0823	0.0688	0.0861
C8	−0.3607	−0.2839	−0.3661	−0.2772
C9	0.7715	0.7520	0.7856	0.7671
C10	−0.7044	−0.7105	−0.7012	−0.7092
C11	0.5695	0.4078	0.6264	0.4264
C12	−0.7710	−0.7552	−0.7730	−0.7591
O13	−0.5557	−0.5003	−0.6337	−0.5749
O14	−0.5035	−0.4972	−0.5135	−0.5129
O15	−0.6941	−0.6670	−0.7363	−0.7160
O16	−0.6013	−0.6662	−0.6138	−0.6840
H17	0.2674	0.2834	0.2506	0.2466
H18	0.2423	0.2381	0.2697	0.2838
H19	0.2674	0.2831	0.2509	0.2745
H20	0.2480	0.2465	0.2536	0.2530
H21	0.2650	0.2634	0.2644	0.2653
H22	0.2609	0.2642	0.2943	0.3108
H23	0.5274	0.5233	0.5269	0.5217
H24	0.2580	0.2460	0.2510	0.2420
H25	0.2600	0.2470	0.2840	0.2750
H26	0.2700	0.2690	0.2650	0.2660

**Table 5:** Intra-molecular and inter-molecular HB lengths (in Å) of LC and LCH molecules at S_0_ and S_1_ states.

Hydrogen bonds	LC		LCH	
	S_0_	S_1_	S_0_	S_1_
O_15_-H_23_·····O_16_= C_11_ / O_16_-H_23_·····O_15_=C_1_	1.569	1.571	1.520	1.599
C_11=_O_16_·····H_32_- O_30_	-	-	2.675	2.070
C_1_= O_15_·····H_29_- O_27_	-	-	1.935	1.865
C_9_ =O_13_····· H_37_- O_36_	-	-	1.979	2.002
C_9_=O_13_·····H_35_- O_33_	-	-	1.872	1.924
O_30_-H_31_·····O_27_– H_29_(WB)	-	-	1.814	1.843

**Table 6:** Dihedral angles -A(°) across intra-molecular and inter-molecular HB’s of LC and LCH at S_0_ and S_1_ states.

Hydrogen bonds	LC		LCH	
	S_0_	S_1_	S_0_	S_1_
O_15_-H_23_····· O_16_= C_11_ / O_16_-H_23_·····O_15_=C_1_	−0.3	0.7	4.2	−10.6
C_11=_O_16_····· H_32_- O_30_	-	-	−36	−52.8
C_1_= O_15_ ·····H_29_- O_27_	-	-	48.7	53.7
C_9_ =O_13_·····H_37_- O_36_	-	-	−0.2	−1.5
C_9_=O_13_·····H_35_- O_33_	-	-	19.2	9.2
O_30_-H_31_·····O_27_– H_29_(WB)	-	-	11.3	32.5
